# An Information-Theoretic Framework for Uncertainty Quantification and Sensitivity Analysis in UWB Fuze Transmitters

**DOI:** 10.3390/s26041164

**Published:** 2026-02-11

**Authors:** Yanbin Liang, Kaiwei Wu, Bing Yang, Shijun Hao, Zhonghua Huang

**Affiliations:** School of Mechatronical Engineering, Beijing Institute of Technology, Beijing 100081, China; 3120195127@bit.edu.cn (Y.L.); wukaiwei@bit.edu.cn (K.W.); 3120215164@bit.edu.cn (B.Y.); 3120215165@bit.edu.cn (S.H.)

**Keywords:** uncertainty quantification, UWB fuze transmitter, feature interaction information

## Abstract

Component tolerances in Ultra-Wideband (UWB) fuze transmitters inevitably induce waveform distortions, which propagate through the signal chain to degrade system-level tactical performance, specifically detection range and ranging resolution. Addressing the lack of quantitative mechanisms linking component variations to operational effectiveness, this study proposes an information-theoretic sensitivity analysis framework. First, we establish a physics-based mathematical model of the transmitter and rigorously validate it against circuit-level simulations to characterize the step recovery diode’s transient response under tolerance disturbances. Second, we employ the Latin Hypercube Sampling-Information Entropy (LHS-IE) method to quantify the uncertainty propagation from parameters to pulse features. Crucially, we introduce Feature Interaction Information (FII) to decode the nonlinear coupling between components. Our results reveal a strong Amplifying effect driven by inductance tolerances, where the interaction between parameters amplifies the joint uncertainty of amplitude and width beyond their individual impacts. Conversely, load resistance primarily dictates amplitude uncertainty as an independent linear factor. The proposed framework provides a theoretical basis for converting traditional tolerance design into an entropy-driven precision allocation strategy, ensuring system robustness under manufacturing constraints.

## 1. Introduction

Proximity fuzes are electronic devices that trigger signals at preset distances via radio frequency, laser, ultra-wideband (UWB), or millimeter wave (MMW) technologies [1]. Owing to their exceptional range resolution, superior penetration capability, and inherent low probability of intercept/low probability of detection (LPI/LPD) characteristics [2,3,4], UWB fuzes have emerged as a pivotal technology for modern precision-guided munitions, anti-armor systems, and fuze systems operating in complex electromagnetic environments.

Current efforts to enhance fuze performance primarily focus on receiver optimization and advanced signal processing algorithms [5,6,7,8,9,10]. However, the quality of the transmitted signal itself remains a critical yet often overlooked aspect

Furthermore, prior studies have established foundational work on UWB transmitter design, including component modeling and analysis under ideally controllable parameters [11,12], as well as detailed circuit behavior analysis under specific component operating conditions in related fields [13,14]. In the broader field of electronic circuit design, well-established global sensitivity analysis methods, such as Monte Carlo simulations and variance-based approaches (e.g., Sobol indices), are routinely employed to assess the impact of parameter variations on system outputs.

However, a critical yet often overlooked aspect is the quality of the transmitted signal itself. Existing studies typically assume that fuzes can generate and radiate ideal, high-fidelity UWB pulses. In practical engineering implementations, constrained by nonlinearities in high-power RF transmission chains, limited instantaneous bandwidth, frequency response of antennas and feed networks, and transient responses of high-speed switching devices, emitted UWB pulses inevitably suffer from waveform distortion, amplitude fluctuation, temporal jitter, and spectral broadening or distortion.

These studies often assume ideally controllable parameters. Yet inherent physical constraints exist: batch-to-batch variations in SRD junction capacitance, temperature drift of inductive components, and precision deviations of load resistors [15]. These component-level parametric tolerances couple through circuit networks, becoming fundamental sources of pulse distortion that directly threaten stringent requirements for time-amplitude joint precision [16,17,18,19]. Consequently, while pursuing cutting-edge echo processing techniques, systematically optimizing the transmitted signal quality of UWB fuzes is essential to overcome current performance bottlenecks and achieve a leap in reliability.

This highlights a specific, under-explored challenge in the context of UWB fuzes: the pressing need for tolerance-aware modeling. The unique requirement for fuze design lies in quantifying the impact of component-level variations on multivariate pulse quality—specifically, the simultaneous stability of amplitude and width—which is paramount for system-level performance robustness. Although prior studies have investigated component modeling [20,21,22], they typically operate under the assumption of ideally controllable parameters. Furthermore, traditional sensitivity analysis methods (e.g., variance-based approaches) and even advanced multi-response optimization frameworks [23] are inadequate for specifically capturing the nonlinear and coupled effects of parametric tolerances on multiple pulse characteristics, such as the Amplifying or Dampening interactions between amplitude and width. Critically, this distortion cannot be eliminated by backend signal processing, forming a hidden bottleneck limiting detection consistency.

Against this backdrop, we identify a clear gap in the literature: a dedicated framework for analyzing the joint uncertainty of key pulse features under parameter tolerances in UWB fuzes is lacking. Existing methods are not tailored to decipher the complex, interactive nature of uncertainty propagation that is critical for fuze reliability.

In recent years, information-theoretic approaches have gained significant traction for uncertainty quantification in complex engineering systems. For instance, Yun et al. established an efficient moment-independent sensitivity analysis framework based on the principle of maximum entropy [24], while Wang et al. successfully applied information entropy to quantify the global sensitivity of structural seismic demands [25]. Furthermore, Li et al. advanced computational efficiency by integrating multi-fidelity surrogate models with global sensitivity analysis [26]. While these studies demonstrate the efficacy of entropy-based metrics in capturing global sensitivities beyond variance-based limitations, they predominantly focus on ranking the magnitude of parameter influence. However, a critical gap remains in explicitly quantifying the ‘nature’ of parametric coupling—specifically, distinguishing between synergistic (constructive) and Dampening (destructive) interactions. In the context of UWB transmitters, where component tolerances often trigger complex coupled distortions, understanding these specific interaction mechanisms is as crucial as identifying the main effects.

To address this fundamental bottleneck, this study introduces a novel sensitivity analysis framework based on information entropy (IE) to elucidate the mechanisms of uncertainty propagation. Unlike traditional approaches, the IE framework concurrently captures joint uncertainty in amplitude, pulse width, and other features. We present our LHS-IE (Latin Hypercube Sampling-Information Entropy) method not just as another sensitivity tool, but as a specific solution designed to overcome the limitations of independent tolerance assumptions. It establishes a parametric-to-entropy mapping to quantify how physical-layer distortion erodes system information capacity. Furthermore, we innovatively employ feature interaction information (FII) to characterize the synergistic or Dampening effects between key pulse features, revealing hidden parametric interactions to inform tolerance allocation strategies. This entropy-driven tolerance allocation framework transcends conventional statistics, providing an information-theoretic basis for core component selection and system margin design [27,28,29,30], thereby fundamentally enhancing transmitted signal fidelity and system robustness.

Subsequent sections are structured as follows: Section 2 establishes the tolerance mathematical model for the UWB fuzes transmitters, focusing on the Step Recovery Diode (SRD) pulse generator circuit. Section 3 elaborates on the novel LHS-IE sensitivity analysis model. Section 4 details the hardware experimental validation, and Section 5 concludes with research outlooks.

## 2. Tolerance-Based Mathematical Model of UWB Fuze Transmitter

### 2.1. Operational Principle and Impact Analysis of Pulse Features on System Performance

**Figure 1:**
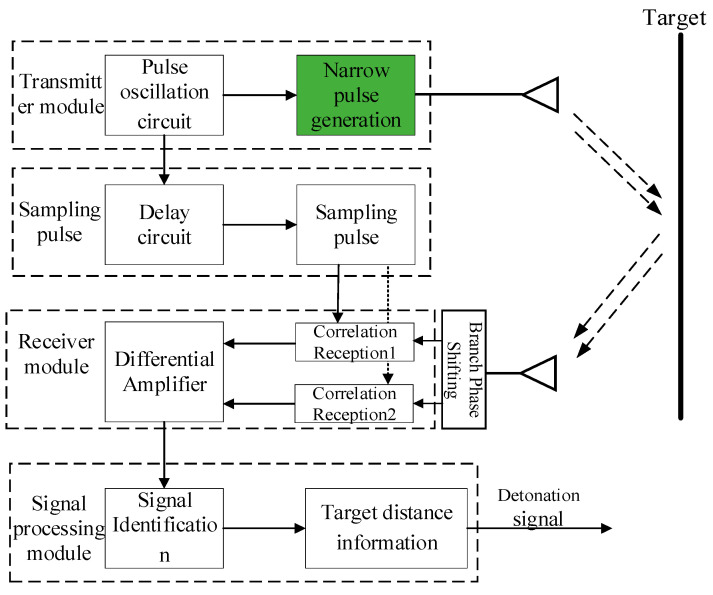
UWB fuze Block Diagram.

The UWB fuzes system incorporates four essential functional modules: pulse transmitter module, antenna module, correlation receiver module, and signal processing module [6]. Within the transmitter module, a pulse oscillation circuit drives the narrow-pulse generation unit to radiate nanosecond-level RF signals toward the target via the antenna module. Simultaneously, a delay circuit precisely regulates sampling pulse timing to establish an accurate range-gating mechanism. The receiver module utilizes coherent reception techniques to capture target echo signals. When the projectile-target distance enters the preset detonation altitude range, sampling pulses trigger signal acquisition followed by correlation computation. Upon processing signals from the receiver, the signal processor extracts target range via threshold comparison algorithms. If the target distance satisfies the preset height-of-burst requirement, it triggers the detonation signal. The schematic block diagram of the UWB fuzes principle is shown in Figure 1 [13].

To quantitatively link the transmitter’s waveform quality to the tactical performance of the UWB fuze, we first establish the fundamental detection model. Unlike communication systems governed by the Friis transmission equation (one-way path loss), a proximity fuze operates as a monostatic radar system. Therefore, its received echo power $P_{r}$ is governed by the Radar Range Equation, which accounts for the two-way propagation loss and target reflection:

(1) $$P_{r}=\frac{P_{t}G_{t}G_{r}\lambda^{2}\sigma }{{\left(4\pi \right)}^{3}R^{4}},$$

where $P_{t}$ is transmitted power, $G_{t}$ and $G_{r}$ represent transmit and receive antenna gains respectively, λ is the wavelength, σ is the target’s radar cross-section (RCS), and R is the distance between the target and the fuzes.

The transmitted power $P_{t}$ can be further expressed as:

(2) $$P_{t}=F\cdot \int_{0}^{T_{p}}\frac{V{\left(t\right)}^{2}}{R_{\mathrm{eff}}\left(f\right)}dt$$

where F is the pulse repetition frequency, $T_{p}$ represents the pulse duration, V corresponds to the instantaneous voltage across the load, and $R_{\mathrm{eff}}$ signifies the effective load resistance.

Equations (1) and (2) reveal the intrinsic coupling between pulse waveform quality and system-level tactical performance, which can be quantified based on classical radar theory [31]:

- Correlation between Amplitude and Detection Range:

By substituting Equation (2) into Equation (1), it is evident that the transmitted power is proportional to the square of the pulse amplitude ($P_{t}\propto V_{peak}^{2}$). According to the radar range equation [31], the maximum detection range $R_{max}$ is proportional to the fourth root of the transmitted power ($R_{max}\propto \sqrt[{4}]{P_{t}}$). Consequently, the relationship between detection range and pulse peak voltage is derived as: $\;R_{max}\propto {\sqrt{V_{\mathrm{peak}}}}_{max}$. This implies that any tolerance-induced degradation in $V_{peak}$ leads to a square-root reduction in the effective surveillance volume.

- Correlation between Pulse Width and Ranging Precision:

Furthermore, for UWB fuzes utilizing time-domain correlation, the range resolution (ΔR)—defined as the minimum distance to distinguish adjacent targets—is physically constrained by the pulse width ($T_{p}$):$\;\Delta R=\frac{c\cdot T_{p}}{2}$. Where c is the speed of light. Pulse broadening or tail oscillations caused by component tolerances essentially “blur” the echo signal. This deterioration leads to ranging errors and blind zone expansion, which severely impacts the detonation timing precision during the terminal guidance phase.

When pulse characteristics deteriorate beyond a critical threshold, the system experiences significant degradation in target ranging accuracy and operational stability. Maintaining reliable ranging in high-interference environments becomes substantially more challenging. Crucially, during the terminal guidance phase—where precise distance data determines detonation timing—pulse degradation severely reduces confidence in range solutions, thereby directly compromising fuze actuation precision and overall strike effectiveness.

### 2.2. Tolerance-Based Transmitter Model

**Figure 2:**
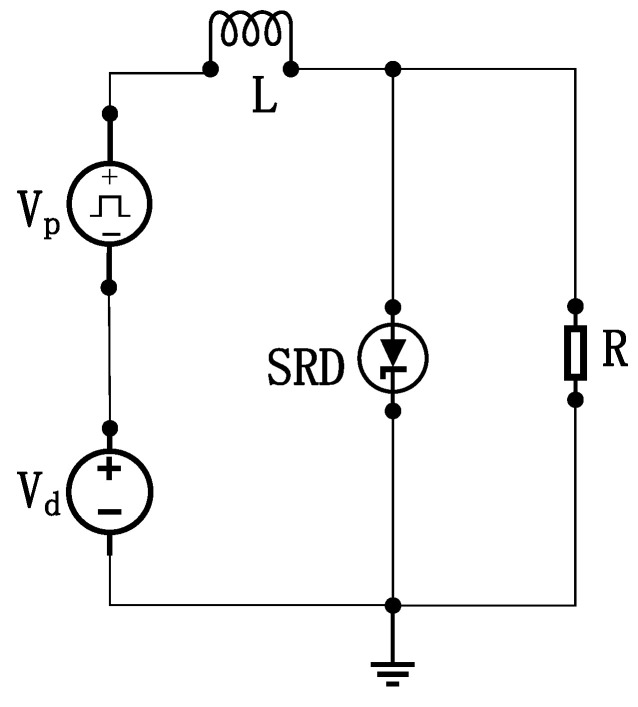
Schematic of the narrow-pulse generation circuit.

The core mechanism of an SRD-based narrow-pulse generation circuit exploits its unique charge storage properties coordinated with the transient response of inductors. Figure 2 illustrates the schematic, where $V_{d}$ is the DC bias supply, $V_{p}$ is the excitation pulse source, L denotes the energy storage inductor, and R represents the load resistance.

During forward bias, the SRD operates in a low-impedance conducting state (approximating a short circuit), with its forward voltage drop near the junction turn-on threshold. Simultaneously, inductor L stores magnetic energy. When the external voltage reverses to initiate a reverse current flow, the SRD initially maintains low-impedance conduction due to its stored minority carriers (charge) not yet being depleted, resulting in a minimal reverse voltage drop.

**Figure 3:**
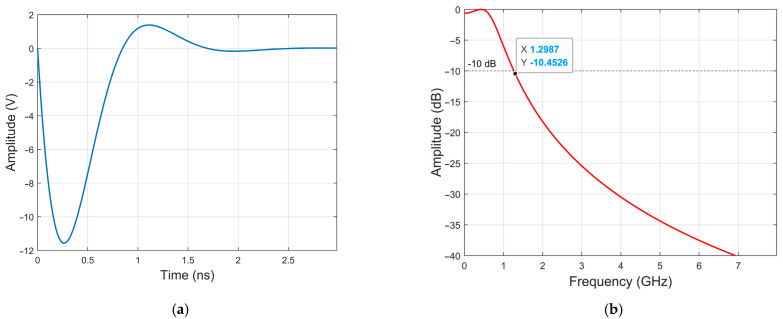
Time-domain and frequency-domain characteristics of the output pulse generated by the UWB fuze; (**a**) time-domain waveform; (**b**) frequency spectrum of the pulse signal.

The critical mechanism arises as stored charge approaches depletion: charge near the PN junction undergoes rapid extraction. Once charge depletion nears a critical threshold, the reverse recovery characteristics of the PN junction trigger an abrupt reduction in junction capacitance, forcing the SRD into an ultra-high impedance state. At this stage, inductor L generates an intense reverse electromotive force (EMF) proportional to $L\left(di/dt\right)$ (Faraday’s law of induction). This high-voltage spike is instantaneously applied across the SRD. The pulse amplitude scales directly with both current decay rate $\left(di/dt\right)$ and inductance value (L). Following pulse emission, the circuit resets and enters the next oscillation cycle with subsequent input signals, thereby cyclically regenerating high-amplitude, ultra-narrow negative pulses. Under ideal conditions, the transmitter generates an UWB pulse signal as shown in Figure 3.

As shown in Figure 3, Figure 3a depicts the time-domain waveform of the fuze transmitter, while Figure 3b shows the frequency spectrum of the pulse signal. The results indicate that the ultra-wideband fuze transmitter achieves a Fractional Bandwidth (FBW) of 200% and an Absolute Bandwidth (B) of 1.2 GHz, thereby complying with the standards set by the U.S. Federal Communications Commission (FCC) and the International Telecommunication Union (ITU) [32,33].

Inevitable physical constraints arise from batch-to-batch dispersion in SRD junction capacitance, temperature drift of inductors, and precision deviations of load resistors. These component-level parametric tolerances couple through the circuit network, forming fundamental distortion sources that induce pulse waveform degradation. Such distortions directly threaten the stringent system requirements for time-amplitude joint precision. The equivalent circuit model considering these tolerances is illustrated in Figure 3.

To accurately assess the effect mechanisms of component tolerances on pulse waveform quality and establish a theoretically rigorous basis for subsequent parametric optimization, this section develops a mathematical model of the narrow-pulse generation circuit that explicitly incorporates component tolerances. Leveraging circuit theory principles, we investigate the dynamic responses of the circuit across different SRD operational states and conduct in-depth analysis of how parameter deviations propagate to the output pulse’s temporal characteristics via circuit dynamic processes.

#### 2.2.1. Modeling of Forward Bias Stage

**Figure 4:**
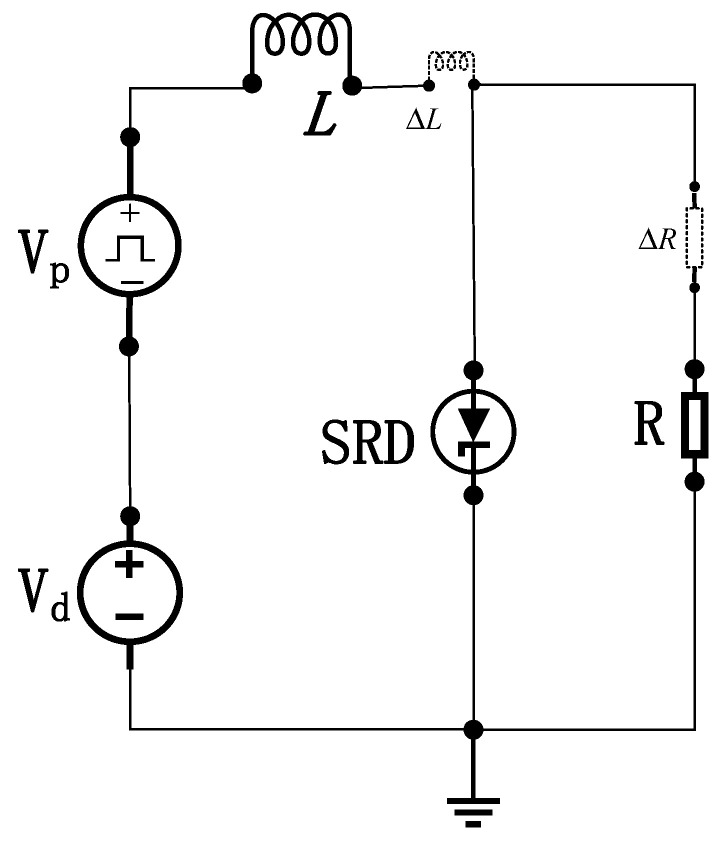
Equivalent circuit incorporating parametric tolerances.

During the pulse intervals of excitation source $V_{p}$, the bias voltage $V_{d}$ maintains the SRD in forward conduction. Typically packaged in surface-mount technology (SMT), the SRD operates under forward bias during this phase. Figure 4 illustrates the equivalent circuit of the narrow-pulse generation circuit under SRD forward bias [3].

**Figure 5:**
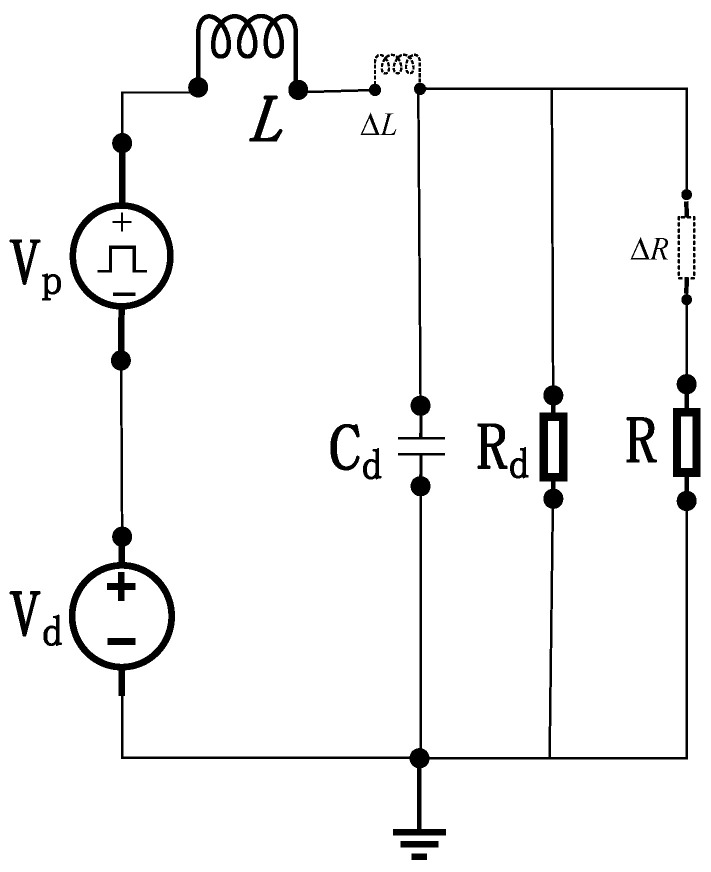
Equivalent circuit for narrow-pulse generation under SRD forward bias.

In Figure 5, $C_{d}$ denotes the diode diffusion capacitance, and $R_{d}$ represents the diode diffusion resistance. Owing to parametric deviations in components, the actual values of the inductor and load resistor deviate from their nominal values. Thus, ΔR and ΔL denote the parametric deviations induced by external conditions and intrinsic component variations.

Under forward bias, the SRD is modeled as a parallel combination of capacitance $C_{d}$ and resistance $R_{d}$, constituting the diode admittance. Here, $C_{d}$ denotes the diffusion capacitance, and $R_{d}$ represents the diffusion resistance. The equivalent parallel resistance of $R_{d}$ and the load resistance $R_{L}$ is mathematically expressed as:

(3) $$R_{L}=R_{d}∥\left(R+\Delta R\right)=\frac{R_{d}\left(R+\Delta R\right)}{R_{d}+R+\Delta R},$$

**Figure 6:**
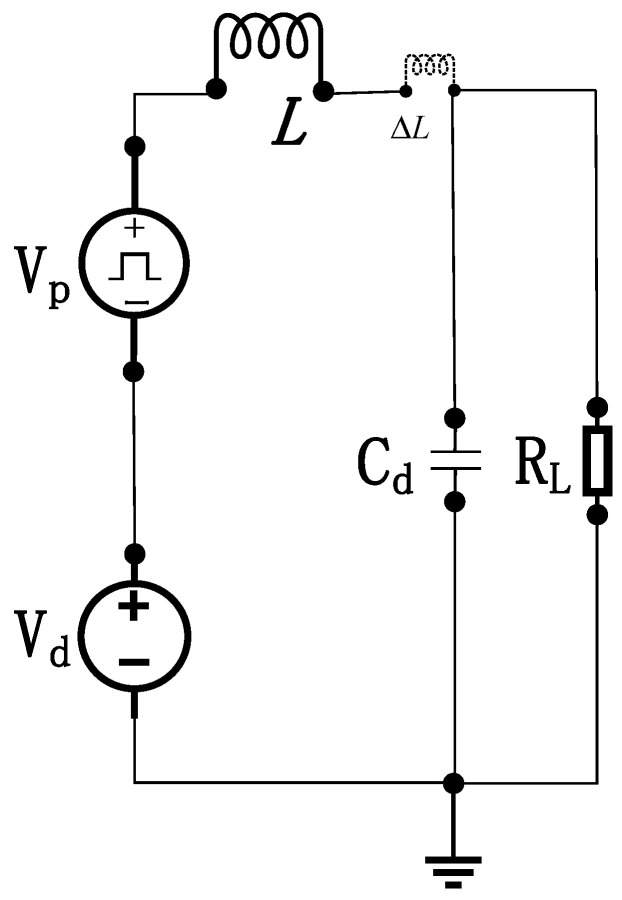
Simplified equivalent circuit for narrow-pulse generation.

Based on the derived equation, the circuit can be further simplified into the model illustrated in Figure 6, showing the equivalent configuration after admittance transformation.

During pulse intervals where $V_{p}=0$, the governing equation derived from Figure 6 is:

(4) $$\left\{\begin{array}{c} L_{\delta }\frac{di_{L_{\delta }}}{dt}+V_{C_{d}}=V_{d}\\ C_{d}\frac{dV_{C_{d}}}{dt}+\frac{V_{C_{d}}}{R_{L}}=i_{L_{\delta }} \end{array},\right.$$

In Equation (4), $L_{\delta }=L+\Delta L$ (where $L_{\delta }$ denotes the actual inductance value), reorganizing yields Formula (5):

(5) $$-R_{L}C_{d}L_{\delta }\frac{d^{2}i_{L_{\delta }}}{dt^{2}}+V_{d}-L_{\delta }\frac{di_{L_{\delta }}}{dt}=R_{L}i_{L_{\delta }},$$

The energy storage capacity of the transmitter is determined by the peak current accumulated in the inductor during the forward bias phase. By solving the differential Equation (5) with the initial condition $i_{L_{\delta }}\left(0^{+}\right)=I_{0}$, the solution to the governing differential equation is:

(6) $$i_{L_{\delta }}\left(t\right)=\frac{V_{d}}{R_{L}}+e^{-\alpha t}\left[\left(I_{0}-\frac{V_{d}}{R_{L}}\right)\cos\beta t+\frac{1}{\beta }\left(\frac{V_{d}}{L_{\delta }}-\alpha \left(I_{0}-\frac{V_{d}}{R_{L}}\right)\right)\sin\beta t\right],$$

where $\alpha =\frac{1}{2R_{L}C_{d}}$ and $\beta =\sqrt{\frac{1}{L_{\delta }C_{d}}-\alpha^{2}}$.

#### 2.2.2. Modeling of Reverse Recovery Stage

**Figure 7:**
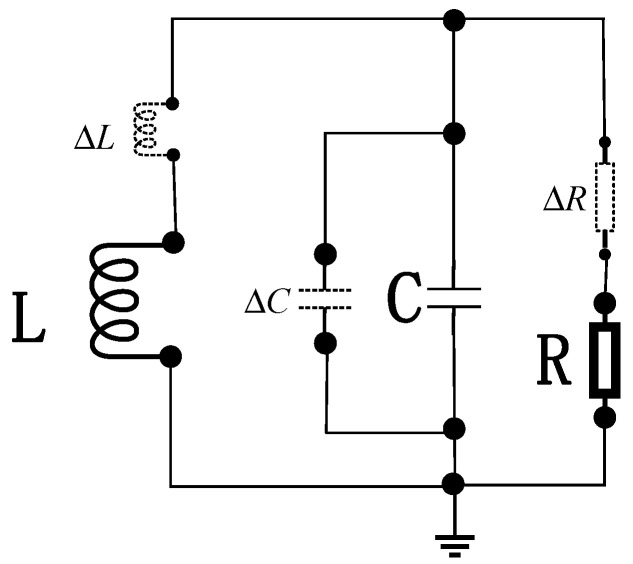
Equivalent Circuit for Narrow-Pulse Generation under SRD Reverse Bias.

When pulse source $V_{p}\;$ generates an excitation pulse, the stored charge in the SRD initiates discharge. Upon depletion of the diode’s stored charge, the inductor current rapidly decreases from its peak value, inducing a high-voltage reverse electromotive force (EMF) spike across the inductor. The equivalent circuit for narrow-pulse generation under SRD reverse bias is shown below in Figure 7.

As indicated in the diagram, C represents the reverse-biased barrier capacitance of the SRD. The governing current and voltage equations for the circuit are given by:

(7) $$\left\{\begin{array}{c} C_{b}\frac{dV_{R_{L}}}{dt}+\frac{V_{R_{L}}}{R_{L}}+i_{L_{\delta }}=0\\ L_{\delta }\frac{di_{L_{\delta }}}{dt}=V_{R_{L}} \end{array}\right.,$$

In Equation (7), $C_{b}$ denotes the post-deviation capacitance value of the diode’s reverse-biased barrier capacitance, expressed as $C_{b}=C+\Delta C$. $R_{L}$ represents the post-deviation resistance value of the load resistor incorporating ΔR, while $L_{\delta }$ indicates the inductance value accounting for excitation inductance deviation ΔL. Reorganizing these terms yields the governing differential Equation (8):

(8) $$L_{\delta }C_{b}\frac{d^{2}i_{L_{\delta }}}{dt^{2}}+\frac{L_{\delta }}{R_{L}}\frac{di_{L_{\delta }}}{dt}+i_{L_{\delta }}=0,$$

Solving this equation yields the inductor current $i_{L_{\delta }}\left(t\right)$ and the voltage across the load resistor $V_{R_{L}}\left(t\right)$, expressed respectively as:

(9) $$\left\{\begin{array}{c} i_{L_{\delta }}\left(t\right)=I_{1}e^{-\alpha^{\prime }t}\left(\cos\beta t+\frac{\alpha^{\prime }}{\beta^{\prime }}\sin\beta^{\prime }t\right)\\ V_{R_{L}}\left(t\right)=-\frac{I_{1}L_{\delta }\beta^{\prime }}{\sqrt{1-\xi }}e^{-\alpha^{\prime }t}\sin\beta^{\prime }t \end{array}\right.,$$

In Equation (9), $\alpha \prime =\frac{1}{2R_{L}C_{b}}$, $\beta \prime =\sqrt{\frac{1}{L_{\delta }C_{b}}-\alpha \prime^{2}}$, $\xi =\frac{1}{4R_{L}^{2}C_{b}/L_{\delta }}$, where $I_{1}\;$ denotes the initial inductor current during SRD reverse bias, determined by the inductor current in the forward-bias phase. As this expression describes the transient process after the SRD enters reverse bias, $I_{1}$ is governed by the circuit state in the preceding forward-bias admittance phase, serving as the initial condition for the reverse-bias stage.

At the onset of reverse bias, the SRD completes its transition from conduction to cutoff, fixing $I_{1}$ independently of reverse-bias parameters ($C_{b}$, $L_{\delta }$, $R_{L}$). Consequently, this study focuses on elucidating the influence mechanisms of $C_{b}$, $\;L_{\delta }$ and $R_{L}$ on pulse waveform characteristics.

### 2.3. Model Validation and Discussion

To ensure the physical rigor of the derived mathematical model [Equations (3)–(9)] and justify its use for subsequent massive sampling analysis, we performed a comprehensive validation through a three-way comparison. This involved circuit-level simulations using PSpice software (version 17.4, OrCAD 2022) and independent experimental measurements. The nominal values of all components, their tolerance ranges, and the detailed PSpice simulation settings are comprehensively provided in Appendix A (Table A1 and Table A2) to guarantee the full reproducibility of all results.

**Figure 8:**
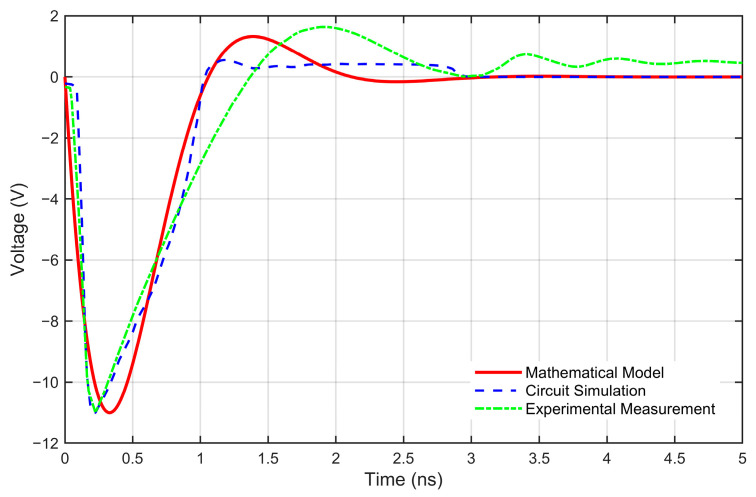
Comprehensive validation of the mathematical model against circuit simulation and experimental measurement.

The integrated comparison is presented in Figure 8, which plots the waveforms from three sources: the proposed mathematical model (red solid line), the SPICE circuit simulation (blue dashed line with square markers), and the experimental measurement (green dotted line with circular markers). This unified visualization allows for a direct and unambiguous assessment of accuracy across the entire modeling chain.

A comparison between the proposed mathematical model and the circuit-level simulation (PSpice) results, as shown in Figure 8, demonstrates a high degree of consistency in core characteristics such as pulse width and amplitude, effectively validating the model’s capability to capture the essential physics. The minor local discrepancies observed primarily stem from necessary idealizations introduced to derive an analytical solution. These include: (1) the assumption of ideal components, neglecting parasitic effects inherent in resistors, inductors, and capacitors at high frequencies; (2) the linearized approximation of the highly nonlinear switching behavior of the step recovery diode (SRD); and (3) the assumption of lossless transmission lines, omitting conductor losses and dielectric dispersion. While these simplifications introduce quantitative deviations, the established model reliably captures the influence of parameter variations on pulse characteristics and provides an efficient theoretical foundation for the subsequent parametric sensitivity analysis of the system.

To assess the model’s predictive capability in real-world conditions, it is further compared with independent experimental measurement data. The results indicate that the mathematical model and the experimental data are fundamentally aligned in key pulse characteristics, notably in the pulse initiation timing and the overall pulse envelope. This confirms the model’s ability to predict the primary behavioral trends of the physical system. The observed deviations between the model and the measurement are attributed to two distinct sources: first, the idealized assumptions of the mathematical model itself (as mentioned above); and second, inherent real-world variabilities not fully captured even by the SPICE simulation, such as board-level parasitic inconsistencies, and measurement system noise.

**Figure 9:**
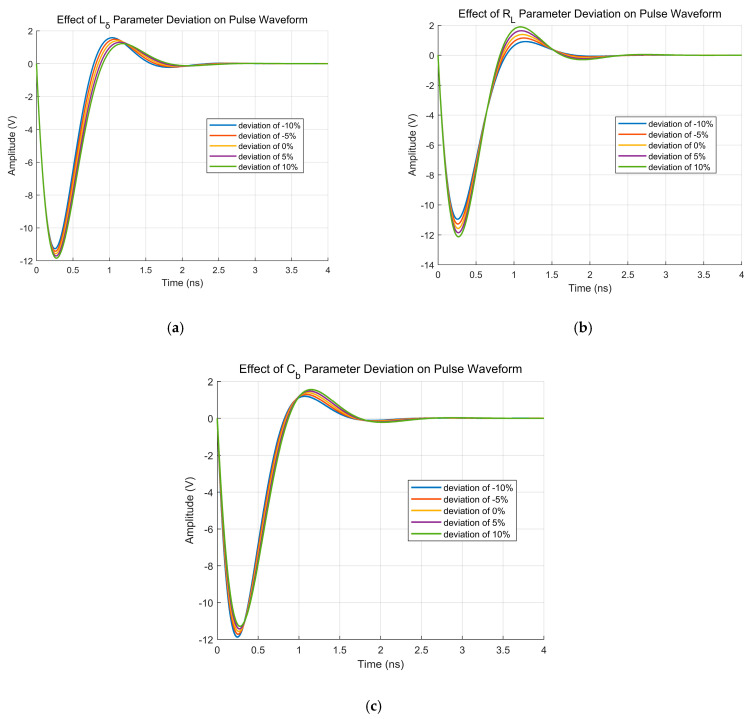
Parametric Sensitivity Analysis of Pulse Waveforms to Component Tolerances: (**a**) $L_{\delta }$ parameter Deviation; (**b**) $R_{L}$ parameter Deviation; (**c**) $C_{b}$ parameter Deviation.

Simulations were performed with nominal parameter values under ±10% and ±5% deviation conditions. The simulation results are shown in Figure 9.

As evidenced by Figure 9, parametric deviations in components induce distinct variations in pulse waveforms, primarily manifested in pulse amplitude and pulse width. Crucially, different parameters exert divergent impacts on these characteristics.

## 3. LHS-IE-Based Sensitivity Analysis

### 3.1. Computational Framework and Discretization Strategy

The proposed framework quantifies the global sensitivity of pulse features to component tolerances using Mutual Information (MI). Based on Shannon’s information theory [34], the MI between a stochastic parameter $X_{i}$ and the output response Y measures the expected reduction in the uncertainty of Y given the knowledge of $X_{i}$. It is mathematically defined as:

(10) $$I\left(X_{i};Y\right)=H\left(Y\right)-H\left(Y|X_{i}\right),$$

In Equation (10), $H\left(Y\right)$ is the marginal entropy representing the total output uncertainty, $H\left(Y|X_{i}\right)\;$ denotes the conditional entropy, which quantifies the residual uncertainty in Y given knowledge of $X_{i}$.

To quantify the contribution of each component’s tolerance to the overall waveform distortion, we define the normalized sensitivity index $\;S_{i}$ is expressed as:

(11) $$S_{i}=\frac{I\left(X_{i};Y\right)}{H\left(Y\right)}\;\left(0\leq S_{i}\leq 1\right),$$

A higher $S_{i}$ value indicates a greater contribution level of parameter $X_{i}$ to characteristic Y.

In UWB fuze transmitter design, pulse signals are characterized by two critical features: pulse amplitude and pulse width. Since IE quantifies the uncertainty of pulse waveforms, introducing IE enables rigorous quantification of uncertainty propagation in these core features induced by component parametric variations.

**Figure 10:**
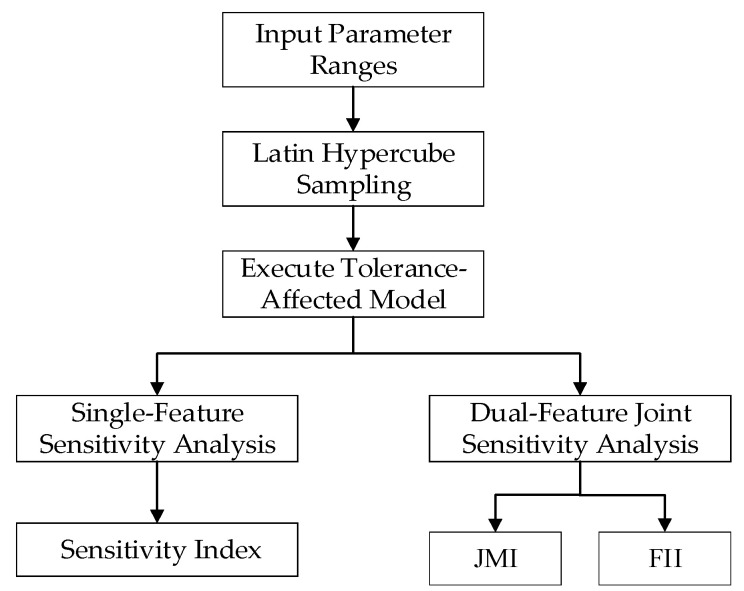
Schematic Diagram of LHS-IE Sensitivity Analysis Framework.

This study proposes a novel LHS-IE framework—integrating Latin LHS and IE theory—for parametric sensitivity analysis. Its essence lies in leveraging information-theoretic metrics to quantify uncertainty propagation mechanisms from component tolerances to UWB pulse characteristics. Figure 10 illustrates the schematic diagram of this methodology.

Utilizing LHS, this method generates N sets of high-dimensional uniformly distributed samples within the component tolerance space. By employing stratified random permutation, it ensures parameter independence, overcoming the curse of dimensionality inherent in traditional Monte Carlo sampling and achieving efficient coverage of the parameter space.

Through single-feature parametric sensitivity analysis and dual-feature joint sensitivity analysis, the framework establishes a hierarchical evaluation system. We innovatively employ Feature Interaction Information (FII) to precisely quantify nonlinear synergistic or Dampening effects between parameters, revealing the physical essence of amplitude-pulse width trade-offs.

The framework models comprehensive time-frequency uncertainty via joint entropy $H\left(V_{\mathrm{peak}},T_{\mathrm{width}}\right)$, constructing a quantitative mapping chain: Tolerances → Entropy Increase Performance Degradation. This provides the first information-theoretic tolerance optimization toolkit for UWB fuzes, enabling a paradigm shift from empirical design to entropy-driven design.

The selection of an entropy estimation method involves a trade-off between bias, computational cost, and parametric complexity. This study employs the histogram (binning) method. It is recognized that other estimators, such as k-nearest neighbors (k-NN) or kernel density estimation (KDE), can be employed for this purpose.

The choice of the histogram method is motivated by the primary objective of this work: to establish a robust and computationally efficient framework for relative sensitivity ranking, rather than to determine the absolute entropy value. The histogram method possesses a key advantage in this context: it operates with a single, intuitive parameter (the number of bins, *M*), making its behavior highly transparent and its results easily reproducible. The convergence analysis in Section 3.4 demonstrates that for *M* > 15, the relative ranking of the sensitivity indices is stable. This confirms that, for the specific goal of comparative sensitivity analysis, the histogram method provides a robust and sufficient foundation, offering an optimal balance between implementation simplicity and analytical reliability.

### 3.2. Sensitivity Analysis

#### 3.2.1. Single-Feature Parametric Sensitivity Analysis

This framework first focuses on quantifying the independent contributions of parametric variations in components $C_{b}$, $L_{\delta }$, and $R_{L}$ within tolerance ranges to pulse peak voltage ($V_{peak}$) and pulse width ($T_{width}$). Leveraging information entropy theory and Latin Hypercube Sampling (LHS), this section establishes a single-feature parametric sensitivity analysis framework.

Target Parameters: $C_{b}$,$\;L_{\delta }$,$\;R_{L}$

Output Features:

- Amplitude: $Y_{1}=V_{peak}$
- Pulse Width: $Y_{2}=T_{width}$

To ensure uniform coverage of the parameter space, $C_{b}$,$\;L_{\delta }$, and $R_{L}$ are assumed to follow uniform distributions.

(12) $$X\left\{\begin{array}{c} R_{L}\sim U\left[R\left(1-\delta_{R}\right),R\left(1+\delta_{R}\right)\right],\;\delta_{R}=10\%\\ L_{\delta }\sim U\left[L\left(1-\delta_{L}\right),L\left(1+\delta_{L}\right)\right],\;\delta_{L}=10\%\\ C_{b}\sim U\left[C\left(1-\delta_{C}\right),C_{b}\left(1+\delta_{C}\right)\right],\;\delta_{C}=10\% \end{array}\right.,$$

The sample size for the LHS was set to n=500. This value provides a robust balance between computational efficiency and the reliable estimation of the output distributions and sensitivity indices for our three-parameter model. The LHS procedure is implemented as follows:

1. Determine Feature Dimension L: In the UWB fuze transmitter model, For the UWB fuze transmitter output model (Equation (9)), there exist three random variables $R_{L}$, $\;L_{\delta }$, $C_{b}$ namely, the feature dimension is L=3. An L-dimensional parameter space is thereby constructed;
2. Generate Sample Matrix: the sample size for the LHS was set to n= 500, This value was chosen to ensure robust coverage of the three-dimensional parameter space ($R_{L}$, $L_{\delta }$, $C_{b}$) and stable estimation of the output distributions. Construct an  N×L-dimensional sample matrix S;
3. Stratified Sampling:
  - For each parameter $L_{k}\;\left(k=1,\;2,\;3\right)$, partition the probability space [0, 1] into N equisized intervals:
    (13) $$\left[0,\frac{1}{N}\right),[\left.\frac{1}{N},\frac{2}{N}\right),\ldots ,\left[\frac{N-1}{N},1\right],$$
  - Within each interval, independently generate uniform random variates:
    (14) $$u_{i}^{\left(k\right)}\sim u\left[\frac{i-1}{N},\frac{i}{N}\right],i=1,\ldots ,N,$$
    yielding an N×3 stochastic matrix:
    (15) $$U=\left[\begin{array}{ccc} u_{1}^{\left(1\right)} & \ldots & u_{1}^{\left(3\right)}\\ \vdots & \ddots & \vdots \\ u_{N}^{\left(1\right)} & \ldots & u_{N}^{\left(3\right)} \end{array}\right],$$
  - To suppress artificial spurious correlations between parameters, perform column-wise random permutation on U, ensuring independence of sampled values;
4. Inverse Cumulative Distribution Function (CDF) Transformation

Transform uniform samples U to the target distribution:

(16) $$S_{i,k}=F_{k}^{-1}\left(u_{i}^{\left(k\right)}\right),$$

where $F_{k}^{-1}\left(\cdot \right)$ is the inverse CDF of the k-th parameter. Given our assumption of uniform distributions, for a parameter with a nominal value $x_{0}^{\left(k\right)}$ and a tolerance of ±p%, the inverse CDF transformation is defined by the linear scaling:

(17) $$F_{k}^{-1}\left(U\right)=x_{0}^{\left(k\right)}\left(1-\frac{p}{100}\right)+U\cdot \left(x_{0}^{\left(k\right)}\cdot \frac{2p}{100}\right)$$

This maps the uniform variable U on $\left[0,\;1\right]$ to a value uniformly distributed within the interval $\left[x_{0}^{\left(k\right)}\left(1-\frac{p}{100}\right),\;x_{0}^{\left(k\right)}\left(1+\frac{p}{100}\right)\right]$.

5. Generation of Sample Matrices

The sampling was initialized using a fixed random seed of 40 to ensure the results are perfectly reproducible. The final N×L sample matrix is constructed as:

(18) $$A=\left[a_{1},a_{2}\ldots a_{L}\right]\left(L=3\right),$$

Each row represents a unique set of component parameters.

An independently generated base matrix B is created using the same sampling method. Both A and B are mutually independent. From these, permutation matrices C are derived as follows:

(19) $$\left\{\begin{array}{c} A=\left[a_{1},a_{2}\ldots a_{L}\right]\\ B=\left[b_{1},b_{2}\ldots b_{L}\right]\\ C^{\left(i\right)}=\left[a_{1}\ldots a_{i-1}b_{i}a_{i+1}\ldots a_{3}\right]\\ C^{\left(ij\right)}=\left[a_{1}\ldots b_{i}\ldots b_{j}\ldots a_{3}\right]\\ C^{\left(ijk\right)}=\left[a_{1}\ldots b_{i}\ldots b_{j}\ldots b_{k}\ldots a_{3}\right] \end{array}\right.,$$

**Figure 11:**
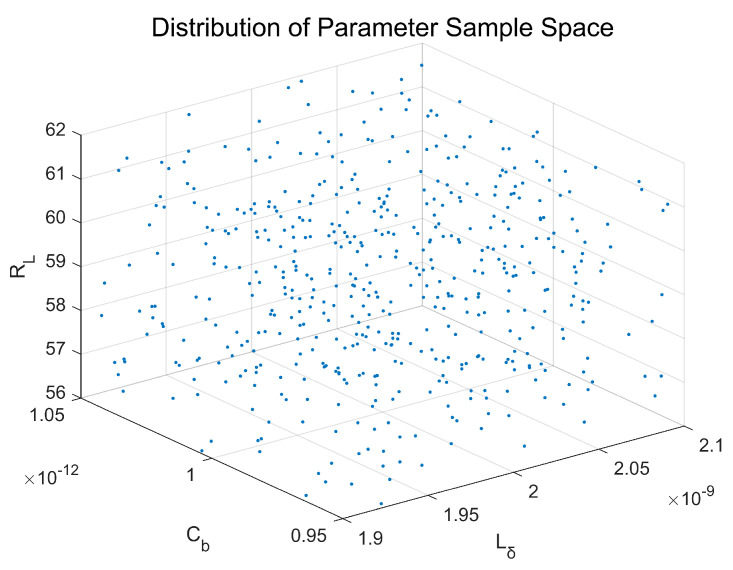
Parameter Distribution of LHS Samples.

The permutation matrices are constructed as follows:

- $C^{\left(i\right)}$: Matrix formed by replacing the *i*-th column of A with the *i*-th column of B;
- $C^{\left(ij\right)}$: Matrix formed by replacing the *i*-th and *j*-th columns of A with the corresponding columns of B, and so forth for higher-order interactions. The distribution of sampling points is shown in Figure 11.

Single-Feature Sensitivity Analysis:

- Feature Extraction

For each output feature $Y_{k}\left(k=1,2\right)$, the following metrics are independently computed:

(20) $$Y_{k}=\left\{\begin{array}{cc} \max\left|V_{R_{L}}\left(t\right)\right| & \left(k=1\right)\\ t_{\mathrm{rise}}-t_{\mathrm{fall}} & \left(k=2\right) \end{array}\right.,$$

**Figure 12:**
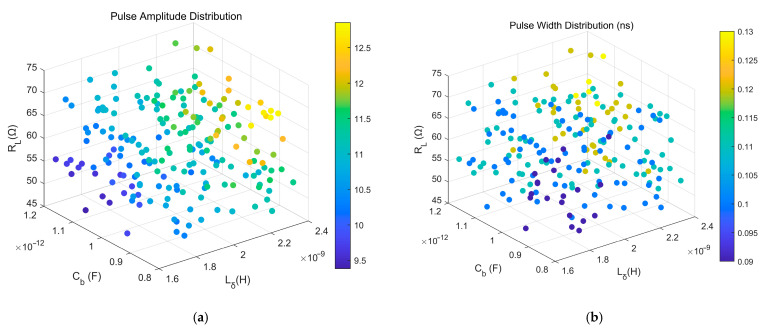
Pulse Feature Distributions: (**a**) Pulse Amplitude Distribution; (**b**) Pulse Width Distribution.

The distributions of generated pulse features are illustrated in Figure 12.

- Computation of Sensitivity Indices
  Discretize $Y_{k}$ into *M* intervals (*M* = 20), The continuous output features $V_{peak}$ and were $T_{width}$ discretized into *M* = 20 bins of equal width across their empirically observed ranges from the simulations. The bin edges were determined based on the minimum and maximum values observed in the initial *n* = 500 sample set.

Compute the marginal entropy $H\left(Y_{k}\right)$ and conditional entropy $H(Y_{k}|X_{j})$:

(21) $$H\left(Y_{k}\right)=-\sum_{m=1}^{M}p\left(y_{m}\right)logp\left(y_{m}\right),$$

(22) $$H\left(Y_{k}|X_{j}\right)=-\sum_{x\in x_{j}}\sum_{y\in y_{k}}p\left(x,y\right)logp(y|x),$$

The $H\left(Y_{k}\right)\;$ quantifies the uncertainty of output feature $Y_{k}$. A more uniform probability distribution of discrete values yields higher entropy, indicating greater sensitivity to parameter variations. Conversely, the $H(Y_{k}|X_{j})$ reflects the control capability of parameter $X_{j}$ over the output feature. A smaller value indicates that $X_{j}$ more significantly reduces the residual uncertainty in $Y_{k}$.

Based on Equation (14), the sensitivity index can be expressed as:

(23) $$S_{j}^{\left(k\right)}=\frac{I\left(X_{j};Y_{k}\right)}{H\left(Y_{k}\right)}=\frac{H\left(Y_{k}\right)-H\left(Y_{k}|X_{j}\right)}{H\left(Y_{k}\right)},$$

The sensitivity index $S_{j}^{\left(k\right)}$ is therefore computed using Equation (23). The quantified results for all studied parameters are summarized in Table 1.

**Figure 13:**
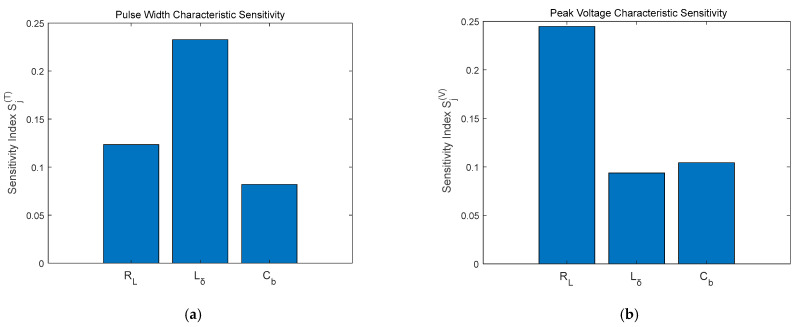
Sensitivity Index: (**a**) Pulse Width; (**b**) Peak Voltage.

Figure 13 quantitatively reveal the differential impact mechanisms of component tolerances on pulse characteristics in UWB fuze transmitters. The load resistance $R_{L}$ primarily governs the uncertainty in peak voltage, while the inductance $L_{\delta }$ significantly modulates pulse width properties. In contrast, the barrier capacitance $C_{b}\;$ exhibits weaker influence on both features, validating its role as a non-critical perturbation source.

#### 3.2.2. Dual-Feature Joint Sensitivity Analysis

Discretize the amplitude ($V_{peak}$) and pulse width ($T_{width}$) into 20 equal-width intervals respectively, and construct a joint probability distribution over a 20 × 20 grid:

(24) $$p\left(v_{m},t_{n}\right)=\frac{N_{m,n}}{N},m=1,\ldots ,20,n=1,\ldots ,20,$$

where $N_{m,n}$ denotes the count of samples falling into grid cell $\left(m,n\right)$.

An analytical framework based on joint entropy is established to quantify the overall uncertainty of dual features. Its mathematical expression is:

(25) $$H\left(V_{\mathrm{peak}},T_{\mathrm{width}}\right)=-\sum_{v,t}p\left(v,t\right)logp\left(v,t\right),$$

The Joint Mutual Information (JMI) quantifies the overall contribution of parameter $\;X_{i}\;$ to the dual-feature system, expressed as:

(26) $$I\left(X_{i};V_{\mathrm{peak}},T_{\mathrm{width}}\right)=H\left(V_{\mathrm{peak}},T_{\mathrm{width}}\right)-H\left(V_{\mathrm{peak}},T_{\mathrm{width}}|X_{i}\right),$$

To decode the nonlinear coupling between component tolerances, we introduce Feature Interaction Information (FII). Unlike simple superposition, parameter interactions can either amplify or suppress the total output uncertainty. To avoid ambiguity with information-theoretic terminology, we classify these interactions based on their physical impact on pulse fidelity:

Dampening:

(27) $$I_{\mathrm{int}}\left(X_{i}\right)=I\left(X_{i};Y\right)-\left[I\left(X_{i};V_{\mathrm{peak}}\right)+I\left(X_{i};T_{\mathrm{width}}\right)\right],$$

where $I\left(X_{i};Y\right)$ denotes the joint mutual information between $X_{i}$ and the bivariate system $Y=(V_{\mathrm{peak}},T_{\mathrm{width}})$, $I\left(X_{i};V_{\mathrm{peak}}\right)$ and $I\left(X_{i};T_{\mathrm{width}}\right)$ represent the mutual information of $X_{i}$ with peak voltage and pulse width, respectively.

Amplifying Interaction ($I_{int}>0$):

This occurs when the joint variation of two parameters creates “new” uncertainty that exceeds the sum of their individual effects. Physically, this represents a destructive scenario for the system, where tolerances reinforce each other to worsen waveform distortion.

Dampening Interaction ($I_{int}<0$):

This indicates information redundancy, where the variation of one parameter partially predicts or offsets the effect of another. Physically, this acts as a buffering mechanism, where coupled tolerances might cancel out, resulting in a more stable output than expected.

Integrating the preceding formulations, the joint sensitivity quantification is expressed as: Amplifying Interaction ($I_{int}>0$):

(28) $$S_{i}^{\mathrm{joint}}=\frac{I\left(X_{i};V_{\mathrm{peak}},T_{\mathrm{width}}\right)}{H\left(V_{\mathrm{peak}},T_{\mathrm{width}}\right)}=\frac{H\left(V_{\mathrm{peak}},T_{\mathrm{width}}\right)\;-\;H\left(V_{\mathrm{peak}},T_{\mathrm{width}}|X_{i}\right)}{H\left(V_{\mathrm{peak}},T_{\mathrm{width}}\right)},$$

The simulation results are presented in Table 2.

As evidenced by Table 2, the joint sensitivity index of the inductor ($S_{L}^{\mathrm{joint}}=0.421$) is statistically significantly higher than that of the load resistor ($S_{R}^{\mathrm{joint}}=0.257$) and barrier capacitance ($S_{C_{b}}^{\mathrm{joint}}=0.194$). This indicates that inductance deviations dominate the comprehensive time-frequency characteristics of the pulse.

Feature Interaction Information further reveals: The inductor exhibits strong positive interaction effects ($I_{\mathrm{int}}\left(L\right)=1.498$), signifying that its tolerance variations exacerbate conflicts between amplitude and pulse width characteristics. In contrast, the load resistor ($I_{\mathrm{int}}\left(R_{L}\right)=\;$0.384) and barrier capacitance ($I_{\mathrm{int}}\left(C_{b}\right)$ = 0.580) also demonstrate positive interaction properties, collectively constituting a synergistic amplification mechanism for pulse distortion.

#### 3.2.3. Determination and Robustness Verification of Key Computational Parameters

To ensure the robustness and reliability of the sensitivity analysis results based on information entropy, the selection of three key computational parameters in this study—the number of Latin Hypercube Samples (N), the number of bins (M), and the number of Bootstrap resamples (B)—was systematically justified. All parameter choices followed the principle of achieving a balance between computational accuracy and efficiency, with their sufficiency being rigorously validated through convergence tests.

The selection of the sample size N aimed to balance computational efficiency with sufficient coverage of the parameter space. This study involves three key variable parameters ($R_{L},\;L_{\delta },\;C_{b}$), constituting a three-dimensional parameter space. The choice of *n* = 500 LHS samples was designed to provide highly uniform coverage of this space, with a sample size far exceeding that required by simple random sampling to achieve comparable precision.

**Figure 14:**
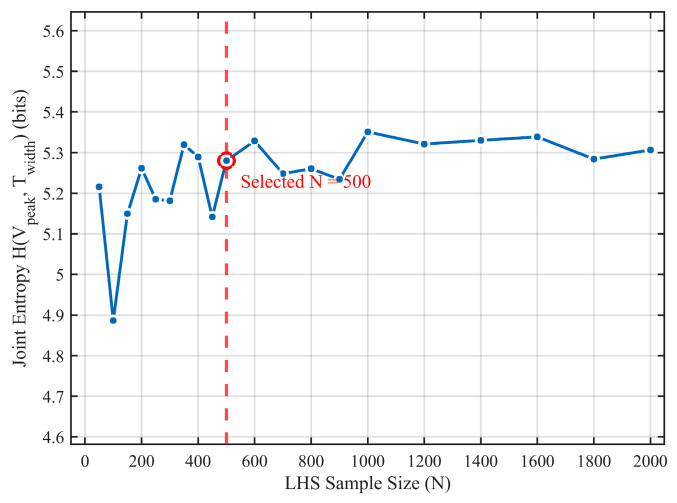
Convergence of the joint entropy estimate.

To validate the sufficiency of *n* = 500, a convergence analysis was performed. As shown in Figure 14, we monitored the change in the joint entropy value of the output pulse features (peak voltage V_peak_, pulse width T_width_) as the sample size N increased from 100 to 2000. The results indicate that when N > 400, the estimated joint entropy value reaches a stable plateau, with fluctuation ranges of less than 2%. Therefore, *n* = 500 provides a robust basis for estimating the probability distributions required for all subsequent analyses.

Having established a robust data foundation with *n* = 500, the next step is to reliably quantify the estimation uncertainty of the sensitivity indices derived from this data. For this purpose, we employ the Bootstrap method with B = 1000 resamples.

This parameter choice is standard practice for generating stable confidence intervals.

For each of the *B* bootstrap samples, the full set of entropy-based sensitivity indices was recalculated. This process generated an empirical distribution for each index, from which the 95% confidence intervals (CIs) were determined as the 2.5th and 97.5th percentiles.

The resulting narrow CIs (presented in Table 1 and Table 2) not only validate the precision of our point estimates but also confirm that *B*= 1000 was sufficient for reliable uncertainty quantification.

The number of bins M determines the granularity of discretization for continuous variables, directly affecting the accuracy of the information entropy calculation. To maintain consistency within the analytical framework, the same number of bins (M = 20) was used for each feature when calculating both single-feature (one-dimensional) entropy and dual-feature joint (two-dimensional) entropy. This approach ensures that all entropy values, from one-dimensional to two-dimensional, are calculated on a unified discretization benchmark. Consequently, differences can be purely attributed to the intrinsic statistical relationships between the features, rather than artificial changes in scale.

**Figure 15:**
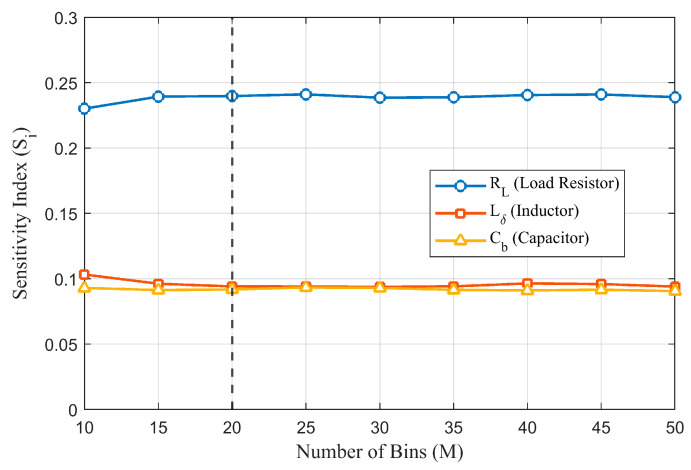
Convergence test of sensitivity indices ($S_{i}$) versus the number of discretization bins (M).

To determine the optimal M value, we performed a convergence test to examine how the sensitivity indices ($S_{i}$) fluctuate when varying the number of bins M from 10 to 50. The results, as illustrated in Figure 15, demonstrate that the parameter sensitivity ranking remains statistically stable, with the maximum relative change for any individual index staying below 5% when M≥15. This confirms that our choice of M=20 is robust against the granularity of discretization and represents an optimal trade-off between resolution and estimation stability.

### 3.3. Dual-Parameter Interaction Analysis

**Figure 16:**
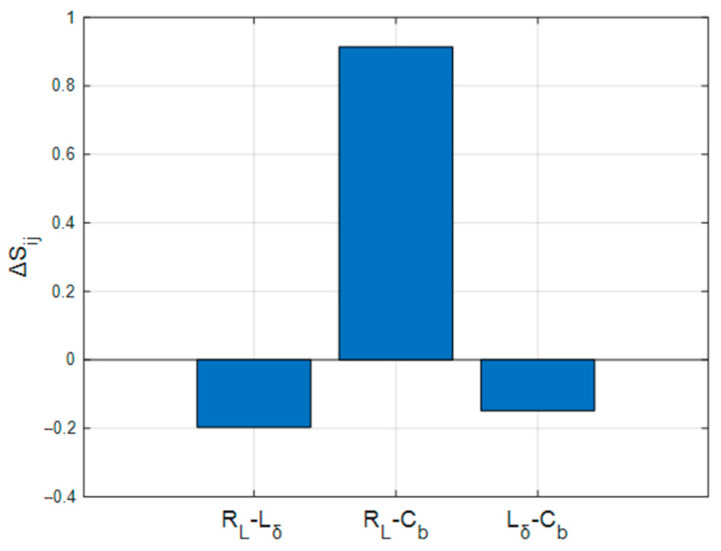
Dual-Parameter Interaction Index.

The aforementioned single-parameter sensitivity analysis has revealed the independent contribution of each component to the system’s overall uncertainty. However, in practical circuits, parametric tolerances of multiple components may deviate simultaneously and interact with each other. Within the information entropy-driven sensitivity analysis framework, the metric $\;\Delta S_{ij}$ is employed to characterize the net impact of dual-parameter interactions on system uncertainty. This metric intrinsically quantifies the synergistic and Dampening effects between parameters, defined as:

(29) $$\Delta S_{ij}=S_{ij}^{\mathrm{joint}}-\left(S_{i}^{\mathrm{joint}}+S_{j}^{\mathrm{joint}}\right),$$

where $S_{ij}^{\mathrm{joint}}=\frac{I\left(\left(X_{i},X_{j}\right);Y\right)}{H\left(Y\right)}$. When $\Delta S_{ij}\;$< 0, Indicates that the coupling effect of dual parameters reduces system uncertainty (synergistic effect), meaning their combined information contribution exceeds the sum of individual contributions, and parameter interactions counteract stochastic disturbances; when $\Delta S_{ij}\;$> 0, Reflects that the parameter combination amplifies system uncertainty (Dampening effect), where their interaction induces additional information loss, i.e., conflicting coupling amplifies disturbances. Simulation results are shown in Figure 16.

As illustrated in Figure 16, the load resistance-inductance combination ($R_{L}$, $L_{\delta }$) exhibits significant synergistic effects ($\Delta S_{ij}=-0.20$). This demonstrates their complementary mechanisms—where $R_{L}$ stabilizes voltage amplitude and $L_{\delta }$ regulates pulse width timing—jointly suppressing time-frequency uncertainty in pulses. Conversely, the load resistance-barrier capacitance combination ($R_{L}$,$C_{b}$) displays intense Dampening effects ($\Delta S_{ij}=0.81$), with their conflict nonlinearly amplifying pulse distortion. The inductance-barrier capacitance pair ($L_{\delta }$, $C_{b}$) shows moderate Amplifying Interaction ($\Delta S_{ij}=-0.17$), reflecting waveform fidelity optimization via energy-time decoupling mechanisms.

This quantitative model reveals non-additive propagation laws of component tolerances, establishing an entropy-driven tolerance allocation criterion for fuze design: exploit relaxed tolerances for synergistic parameters while strictly isolating conflicting ones.

### 3.4. Comparative Validation Against Variance-Based Methods

To evaluate the effectiveness of the information entropy sensitivity analysis framework and validate its conclusions, this section introduces the classical Sobol method as a comparative benchmark. The Sobol method is a global sensitivity analysis technique based on variance decomposition. Its core principle decomposes the total variance of model output into the sum of variance contributions from individual input parameters acting alone and their coupled interactions. This approach systematically quantifies both the main effects of single parameters (first-order sensitivity indices) and the impact of parameter interactions on output uncertainty (higher-order sensitivity indices). As a classical sensitivity analysis tool, the Sobol method has well-established applications across numerous engineering domains.

Sobol Sensitivity Analysis Procedure [29]:

The UWB fuze transmitter model is expressed as $y=V_{R_{L_{\delta }}}$, the component parameter variables—load resistance $R_{L}$, tolerance-incorporated inductance $L_{\delta }$, and barrier capacitance $C_{b}$—are collectively denoted as X. y represents the target variable. Substitute the sample matrix and permutation matrices x into the transmitter model for simulation, yielding the following system outputs:

(30) $$\left\{\begin{array}{c} u_{A}=u_{0}\left(S\right)\\ u_{B}=u_{0}\left(S^{\prime }\right)\\ u_{C^{\left(i\right)}}=u_{0}\left(C^{\left(i\right)}\right)\\ u_{C^{\left(ij\right)}}=u_{0}\left(C^{\left(ij\right)}\right)\\ u_{C^{\left(ijk\right)}}=u_{0}\left(C^{\left(ijk\right)}\right) \end{array}\right.,$$

The Sobol method, based on ANOVA variance decomposition principles, decomposes the output variance into contributions from input parameters and their interactions. For the circuit output $y=u\left(x\right)$, the total variance decomposes as:

(31) $$Var(y)=\sum_{i}V_{i}+\sum_{i<j}V_{ij}+\sum_{i<j<k}V_{ijk}+\ldots +V_{12\ldots p},$$

where

- Var(y): Total model variance;
- $V_{i}$: Variance contribution of the *i-th* parameter;
- $V_{ij}$: Interaction effect variance between parameters i and j;
- $V_{12\ldots p}$: Variance from joint effects of p parameters.

First-order effect variance and first-order sensitivity index are given by:

(32) $${\overset{¯}{V}}_{i}=\frac{1}{N}\sum_{s=1}^{N}u_{A}^{\left(s\right)}u_{C^{\left(i\right)}}^{\left(s\right)}-{\overset{¯}{u}}^{2},$$

(33) $${\hat{S}}_{i}=\frac{{\overset{¯}{V}}_{i}}{\bar{\mathrm{Var}}\left(u\right)},$$

**Figure 17:**
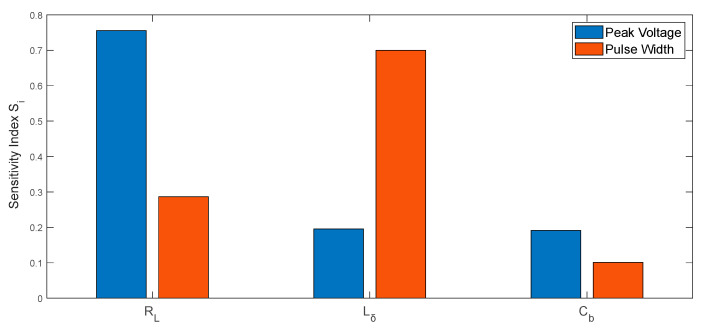
Sobol Sensitivity Indices.

The simulation results are presented in Figure 17.

As evidenced by Figure 17, the Sobol sensitivity analysis corroborates the fundamental reliability of the proposed information entropy framework. A comparative assessment with the entropy-based results in Figure 13 reveals a high degree of consistency in the ranking of parameter main effects for each individual pulse feature. However, the information entropy approach demonstrates superior capability in resolving nonlinear interactions. While Sobol effectively verifies primary parameter effects, its linear decomposition kernel fails to accurately quantify strong nonlinear couplings (e.g., $L_{\delta }$-$C_{b}$ induced pulse oscillations) or non-additive conflicts (e.g., spectral degradation from $R_{L}$-$C_{b}$ combinations).

The entropy framework precisely quantifies component-level mitigation effects on amplitude-pulse width conflicts through Feature Interaction Information, whereas Sobol cannot reveal the physical essence of such parametric Amplifying Interaction. This divergence stems from entropy’s joint probability modeling mechanism: By constructing bivariate distributions $H\left(V_{\mathrm{peak}},T_{\mathrm{width}}\right)$, it directly captures multi-feature coupling uncertainty induced by parameter variations, converting uncertainty propagation into a differentiable information flow model through probability space reconstruction. In contrast, Sobol’s variance decomposition—constrained by linear assumptions—struggles to characterize implicit interaction laws in complex nonlinear systems. Consequently, this study adopts the LHS-IE method for transmitter sensitivity analysis, providing fundamental physical insights for UWB fuze tolerance design.

## 4. Experimental Hardware Verification

To empirically validate the sensitivity analysis results, a hardware test platform for the UWB fuze transmitter was constructed. The experimental protocol was designed to strictly isolate the impact of individual component tolerances following the single-variable control principle.

Key experimental configurations include:

- Baseline Calibration: The transmitter was initially calibrated with high-precision components ±1% (precision) to establish a nominal reference waveform.
- Tolerance Injection: We utilized commercial-grade components to emulate tolerance deviations. Specifically, load resistors $R_{L}$, inductors L and $C_{b}$ with ±10% tolerance were manually substituted into the circuit.
- Statistical Sampling: To mitigate measurement noise and random manufacturing variations, N=50 independent trials were conducted for each tolerance configuration. The output pulses were captured using a high-speed digital oscilloscope (Tektronix DPO7254C, 2.5 GHz) to ensure sufficient temporal resolution.
- Data Processing: The captured waveforms were aligned in the time domain, and statistical metrics (mean, standard deviation) were extracted to quantify the dispersion of pulse features.

**Figure 18:**
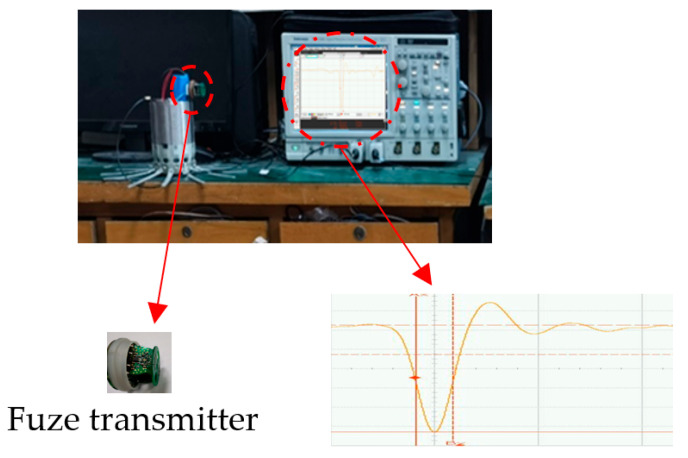
UWB Fuse Transmit Pulse Test.

The test environment configuration is illustrated in Figure 18.

**Figure 19:**
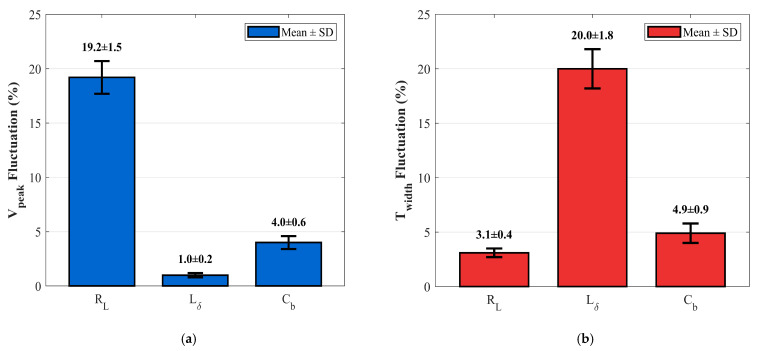
Statistical impact of component tolerances on pulse characteristics. (**a**) Amplitude ($V_{\mathrm{peak}}$) fluctuation. (**b**) Width ($T_{\mathrm{width}}$) fluctuation. (Error bars represent ±1 standard deviation.).

The captured waveforms from the 50 independent trials for each component were processed as described: after time-domain alignment, the key pulse characteristics—amplitude and full width at half maximum—were extracted. The mean value and standard deviation of these parameters were then calculated to quantify the central tendency and dispersion of the performance under each tolerance condition. These statistical results, which directly contrast the ±10% tolerance injection against the ±1% precision baseline, are summarized in Figure 19.

The statistical impact of component tolerances is quantified in Figure 19 using bar charts. Each bar represents the mean percentage deviation of a key pulse parameter, with error bars extending to ±3σ to depict the statistical dispersion of the data. The results reveal distinct sensitivity hierarchies: (a) The load resistance ($R_{L}$) deviation induces a significant amplitude attenuation of approximately 19.2% while maintaining temporal stability, confirming its dominance over the system’s power budget. This amplitude degradation is statistically highly significant (*p* < 0.001, one-way ANOVA). Applying the radar range equation derived in Section 2.1 ($R_{max}\propto \sqrt{V_{peak}}$), this 19.2% amplitude degradation theoretically translates to an approximate 10.6% reduction in the maximum detection range, directly compromising the tactical surveillance volume. (b) Conversely, the inductance (L) tolerance manifests primarily as pulse broadening ($\Delta T_{width}\approx 20.0\%$) with negligible amplitude variation, validating its critical role in determining timing precision and range resolution. The observed pulse broadening is also statistically highly significant (*p* < 0.001, one-way ANOVA). (c) In contrast, the barrier capacitance ($C_{b}$) exhibits minimal impact on both features, as evidenced by the extensive overlap between the baseline and tolerance confidence bands. These empirical findings strongly corroborate the theoretical predictions derived from the proposed LHS-IE sensitivity analysis model.

The hardware verification in Table 3 demonstrates a distinct sensitivity hierarchy, with statistical significance confirming the key effects. The load resistance ($R_{L}$) dominates waveform amplitude stability, inducing a significant and statistically confirmed fluctuation of 19.2%. Conversely, the inductor ($L_{\delta }$) exerts the primary and statistically significant influence on pulse width. These experimental outcomes, rigorously validated by ANOVA, align closely with the LHS-IE model predictions, thereby robustly validating the proposed sensitivity analysis framework.

Entropy-Driven Tolerance Allocation Strategy:

- Based on the sensitivity hierarchy ($S_{L_{\delta }}^{\mathrm{joint}}>S_{R_{L}}^{\mathrm{joint}}>S_{C_{b}}^{\mathrm{joint}}$) and the interaction analysis, we propose a cost-effective tolerance allocation strategy for UWB fuze transmitters. Instead of uniformly assigning expensive high-precision components, designers should adopt a graded precision approach: Identifying the Dominant Factor: The joint sensitivity index (Table 2) identifies the inductor $L_{\delta }$ as the primary source of output uncertainty Therefore, it requires strict control (tolerance ≤ 1%) and low temperature drift coefficient to ensure pulse width stability.
- Exploiting Parameter Interactions: The strong positive interaction $\Delta S_{ij}$ between $R_{L}$ and $C_{b}$ indicates a synergistic effect. This coupling can be leveraged: by selectively pairing components with compensating deviations (e.g., a high-value $R_{L}$ with a low-value $C_{b}$), their individual uncertainties can be made to partially cancel out at the system level.

The case study demonstrates that the optimal strategy is not a uniform tightening of tolerances, but a targeted allocation based on parametric sensitivity and interaction. By investing in a high-precision inductor ($L_{\delta }$) and intelligently leveraging the interaction between the resistor and capacitor ($R_{L,}C_{b}$), the proposed framework achieves a significant performance enhancement efficiently. This provides a concrete, quantitative methodology for robust and cost-effective fuze transmitter design. This study establishes the fundamental relationship under controlled conditions. Future work will incorporate thermal cycling and accelerated aging tests to evaluate long-term reliability under operational environmental stresses.

## 5. Conclusions

To address the challenge of unclear mechanisms through which component tolerances affect time-domain pulse characteristics in ultra-wideband fuze transmitters, this study proposes an information entropy-driven sensitivity analysis framework. By developing a tolerance-integrated mathematical model based on the step recovery diode’s forward/reverse bias equivalent circuits, we systematically investigate the coupled impact of load resistance, inductance, and barrier capacitance deviations on pulse amplitude and width. Key findings include:

First, we establish an ultra-wideband fuze transmitter circuit and develop its equivalent model based on the step recovery diode’s forward and reverse bias characteristics. Through spectral analysis, we verify that the output pulse complies with ultra-wideband spectrum requirements. Our study focuses on addressing the challenge of quantitatively clarifying how component tolerances influence time-domain pulse features.

Second, we propose an information entropy-driven sensitivity analysis framework (LHS-IE) that incorporates two key innovations: (i) LHS-IE method overcomes the linearity assumption of conventional variance-based approaches, enabling us to accurately characterize nonlinear coupling mechanisms among parameters such as inductance and capacitance. (ii) By introducing Feature Interaction Information (FII), we successfully decode Amplifying or Dampening effects between parameters during tolerance propagation in the UWB transmitter.

Third, our analysis demonstrates that the framework effectively identifies critical parameters for precision tolerance control. We validated the approach on an experimental platform, which confirmed a strong correlation between parameter sensitivity and pulse distortion intensity.

The entropy-driven sensitivity analysis framework effectively identifies critical parameters, enabling designers to implement precision tolerance control for high-sensitivity components to block critical failure paths, while relaxing tolerance constraints for low-sensitivity elements to optimize cost structures. This strategy achieves Pareto-frontier optimization between reliability enhancement and cost compression, ensuring core performance stability under tolerance disturbances. Additionally, the multivariate joint information entropy method quantifies Amplifying or Dampening interactions among parameters, overcoming the limitations of independent tolerance assumptions.

Furthermore, the quantitative correlation between pulse quality degradation and system-level performance metrics (e.g., signal-to-noise ratio) is inherently complex due to nonlinear interactions and multi-physics coupling. Therefore, the current study provides a preliminary framework based on information entropy-driven sensitivity analysis, primarily focusing on the impact of parameter tolerances on pulse characteristics. Future work will address multi-physics coupling to establish a direct mapping from pulse quality to system-level performance. This will link component-level tolerances to overall system robustness, facilitating the development of a cross-level reliability model that directly correlates component variations with system performance.

## Figures and Tables

**Table 1:** Sensitivity Index Analysis Results with 95% Confidence Intervals.

	$S_{j}^{\left(V\right)}$	$S_{j}^{\left(T\right)}$
$R_{L}$	0.240 [0.232, 0.248]	0.126 [0.119, 0.133]
$L_{\delta }$	0.095 [0.088, 0.102]	0.226 [0.218, 0.234]
$C_{b}$	0.092 [0.085, 0.099]	0.082 [0.076, 0.088]

**Table 2:** Quantified Joint Sensitivity Indices and Feature Interaction Information with 95% Confidence Intervals.

	$S_{i}^{\mathrm{joint}}$	$I_{\mathrm{int}}\left(X_{i}\right)$
$R_{L}$	0.257 [0.249, 0.265]	0.384 [0.370, 0.398]
$L_{\delta }$	0.421 [0.410, 0.432]	1.498 [1.450, 1.546]
$C_{b}$	0.194 [0.186, 0.202]	0.580 [0.562, 0.598]

**Table 3:** Percentage Fluctuation of Pulse Characteristic Metrics and Statistical Significance.

	$V_{\mathrm{peak}}\;Fluctuation$(%)	$T_{\mathrm{width}}\;Fluctuation$(%)	Statistical Significance $(V_{\mathrm{peak}}$)	Statistical Significance $(T_{\mathrm{width}}$)
$R_{L}$	19.2 ± 1.5	3.1 ± 0.4	*p* < 0.001	NS
$L_{\delta }$	1.0 ± 0.2	20.0 ± 1.8	NS	*p* < 0.001
$C_{b}$	4.0 ± 0.6	4.9 ± 0.9	NS	NS

NS: Not Significant.

## Data Availability

The data are available from the corresponding author upon reasonable request.

## Abbreviations

The following abbreviations are used in this manuscript:

UWB	Ultra-wideband
SNR	Signal-to-noise ratio
RF	Radio frequency
MMW	Millimeter-wave
LHS	Latin Hypercube Sampling
IE	Information entropy
LPI	Low probability of intercept
LPD	Low probability of detection
SRD	Step recovery diode
CDF	Cumulative Distribution Function
JMI	Joint Mutual Information
FII	Feature Interaction Information

## Appendix A

This appendix provides the necessary details to ensure the full reproducibility of the simulation and experimental results presented in the main manuscript. It is structured into three subsections: nominal component parameters, simulation settings, and the hardware test configuration.

### Appendix A.1

The UWB fuze transmitter circuit analyzed in this work is based on the SRD pulse generation topology. The nominal values, tolerances, and key model parameters for all critical components are listed in Table A1. These values define the baseline design and the parametric variations studied.

**Table A1:** Component Parameters and SPICE Model Specifications.

Component	Symbol	Nominal Value	Tolerance
Load Resistor	$R_{L}$	200 Ω	±1% (Baseline), ±10% (Tolerance group)
Energy Storage Inductor	$L_{\delta }$	50 nH	
SRD Barrier Capacitor	$C_{b}$	1 pF	
DC Bias Voltage	$V_{d}$	15 V	
Excitation Pulse Amplitude	$V_{p}$	10 V	
Excitation Pulse Width	$T_{w}$	10 ns	

### Appendix A.2

All circuit-level simulations and tolerance analyses were performed using the Cadence PSpice simulation environment. The detailed solver settings, crucial for achieving accurate and stable transient analysis of the nonlinear SRD circuit, are summarized in Table A2.

**Table A2:** PSpice Simulation Configuration.

Setting Category	Parameter	Value/Option
Simulation Environment	Software & Version	Cadence PSpice 17.2-2016
Analysis Type	Analysis	Transient (Time Domain)
Simulation Time	Run to Time	50 ns
Solver Settings	Maximum Step Size	10 ps
	Integration Method	Trapezoidal (TRAP)
	Relative Error Tolerance	0.001 (0.1%)

### Appendix A.3

The hardware validation tests were conducted using the following instrumentation and configuration to ensure measurement accuracy and reproducibility. The setup is designed to minimize external noise and loading effects.

**Table A3:** Hardware Test Setup and Instrument Specifications.

Item	Specification
Digital Oscilloscope	Tektronix DPO7254C Bandwidth: 2.5 GHz Sampling Rate: 40 GS/s Input Impedance: 50 Ω
Voltage Probe	Tektronix TPP1000 (Passive) Bandwidth: 1 GHz Input Resistance: 10 MΩ Input Capacitance: 3.8 pF Attenuation: 10:1
DC Power Supply	Keithley 2230-30-1
Pulse Pattern Generator	Tektronix AFG3252
Test Environment	Temperature: 25 ± 2 °C Humidity: 45% ± 10%
